# The association of composite dietary antioxidant index with periodontitis in NHANES 2009–2014

**DOI:** 10.3389/fimmu.2024.1384272

**Published:** 2024-06-24

**Authors:** Zihan Meng, Wenzhuo Zheng, Xiwei Meng, Hui Xu

**Affiliations:** ^1^ State Key Laboratory of Oral Diseases, West China Hospital of Stomatology, Sichuan University, Chengdu, China; ^2^ Department of Pharmacology and Therapeutics, McGill University, Montreal, QC, Canada; ^3^ Department of Orthodontics, West China Hospital of Stomatology, Sichuan University, Chengdu, China

**Keywords:** composite dietary antioxidant index, periodontitis, tooth loss, smoking, National Health and Nutrition Examination Survey

## Abstract

**Background:**

To date, evidence is rare regarding whether and how dietary antioxidants are associated with the risk of periodontitis. This study aimed to investigate the association of composite dietary antioxidant index (CDAI) with periodontitis and tooth loss, using data from the National Health and Nutrition Examination Survey (2009–2014).

**Methods:**

A cross-sectional analysis was conducted using data from 10,067 adults aged ≥30 years who underwent assessments of periodontal health and the 1^st^ day dietary recall. Based on a crude model and three adjusted models, multivariate regressions were used to examine the relationship between CDAI and periodontitis-related measurements including probing pocket depth, clinical attachment loss and tooth loss. Subgroup analyses and the restricted cubic splines plots were applied to examine the association between CDAI ingredients and periodontitis.

**Results:**

For the subjects with high CDAI scores, increased CDAI was associated with significant (*P <* 0.05) reduction of severe periodontitis (odd ratio = 0.663, 95% confidence interval: 0.491–0.896) and increased number of remaining teeth (weighted β[SE] = 1.167[0.211]). However, the protective effect of CDAI on periodontitis vanished (*P* > 0.05) in active smokers and former smokers. There were threshold levels for β-carotene, Vitamin A, C and E intakes where the risk of periodontitis significantly decreased (*P <* 0.05) above these levels.

**Conclusion:**

Increased CDAI was associated with reduced risk of periodontitis and tooth loss for non-smokers. It was recommendable that proper dietary intakes of β-carotene, Vitamin A, C and E would be of benefit for preventive dental care and adjuvant therapies for periodontitis.

## 1 Introduction

Periodontitis is a progressive chronic inflammatory disease that, when left untreated or inadequately treated, can lead to destruction of the tooth-supporting tissue and ultimately loss of teeth (1). It ranked as the sixth most prevalent disease worldwide, with a prevalence of up to 50% (2, 3). Several factors, including global population growth and aging, have contributed to the increased prevalence of severe periodontitis over the past three decades (2). At the meantime, the global burden of periodontitis has remained substantial and even grown over the years (4).

The pathogenic events in periodontitis involve complex interactions between microbial communities and host responses. The susceptibility to periodontitis varies among different populations (3, 5). Increasing evidence has stressed the predominant role of the host’s inflammatory responses in the pathogenesis of periodontitis. Upon microbial challenge, immune cell-mediated oxidative stress propagates the pro-inflammatory signaling and eventually leads to tissue damage (3, 6).

Oxidative stress, characterized by overactivation of reactive oxygen species (ROS) and reduced antioxidant capacity, has been extensively studied as one of the main factors contributing to periodontitis (7). Patients with periodontitis have elevated serum ROS levels, which can be effectively reduced by circulating antioxidant (8). It has been well documented that antioxidant from diet can reduce the body’s oxidative stress and lower the risk of systemic diseases, such as diabetes mellitus and cardiovascular diseases (9, 10). These diseases are accompanied by worsened periodontal health and tooth loosening (11). It is plausible to speculate that antioxidant intake from diet might also impact the prevalence and severity of periodontitis. Specifically, in populations and countries with low accessibility to dental care and high prevalence of periodontitis (2), appropriate micronutrient intake may have a significant effect on the morbidity.

To date, evidence is rare regarding the association of dietary antioxidant and the risk of periodontitis and tooth loss. Vanessa et al. demonstrated a positive linear association between the inflammatory degree of the diet (12), measured via the dietary inflammatory index (DII), and periodontitis. Revealing the link between dietary antioxidant intake and periodontitis might have more significance in diet instructions and adjuvant therapeutic strategies. The composite dietary antioxidant index (CDAI) summarized multiple dietary antioxidants including Vitamins A, C, and E, β-carotene, selenium, and zinc (13). This index was developed based on the aggregate effect of these antioxidants. Several researches demonstrated the antioxidant effects of the individual component (14, 15). A previous study demonstrated that CDAI more precisely captures an individual’s dietary antioxidant profile and reduces misclassification of exposure (16). In this study, we analyzed the National Health and Nutrition Examination Survey (NHANES) database to investigate the potential association between CDAI and periodontitis, hoping to provide reference for public oral health care based on diet instructions.

## 2 Materials and method

### 2.1 Study design and participants

This study used data from the NHANES collected in 2009–2010,2011–2012 and 2013–2014. The data was collected via interviews, physical and laboratory examinations. All of the data in NHANES 2009–2014 were reviewed and approved by the Centers for Disease Control (CDC) and Prevention National Center for Health Statistics Research (NCHS) Ethics Review Board. All of the participants provided a written informed consent. The data derived from edentulous participants or participants with invalid oral examinations or invalid 1^st^ day dietary recalls were excluded.

### 2.2 Dietary assessment

NHANES participants underwent a dietary recall interview collected in-person followed by a full-mouth periodontal examination for those aged 30 years and older at a mobile examination center. The dietary recall interviews were used to collect data on the types and amounts of foods and beverages (including all types of water) consumed during the last 24 hours (midnight to midnight), and to estimate intakes of nutrients, energy and other ingredients from those foods and beverages. The CDAI is a summary score of multiple dietary antioxidants including Vitamins A, C, and E, β-carotene, selenium, and zinc. In the present study, we calculated the weighted average of the data from the 1st 24-hour recall interviews. Then, we calculated the z-score for each micronutrient parameter. The CDAI was calculated using the formula as below:


$$\text{CDAI}=\sum_{\text{i}=1}^{\text{n}=6}\frac{\text{Individual Intake }-\text{ Mean }}{\text{SE}}$$

For all of the participants, the CDAI scores were equally divided into four grades, CDAI-1^st^ to 4^th^, in ascending order of the anti-inflammatory capacity.

### 2.3 Periodontal assessment

Full-month periodontal examination at six sites per tooth was conducted by trained calibrated examiners for participants aged 30 years and older. Participants were eligible for the periodontal assessments if they did not meet any of the health exclusion criteria and had at least one tooth (excluding third molars). Probing pocket depth (PPD) and gingival recession were measured using a HU-Friedy periodontal probe graduated in 2 mm increments. Clinical attachment loss (CAL) was calculated as the difference between PPD and gingival recession (17). The primary outcome was listed as moderate/severe periodontitis according to the recommendations of the Centers for Disease Control and Prevention (18). The secondary outcomes were the number of remaining teeth (excluding third molars), mean PPD and mean CAL measured at the interproximal sites.

### 2.4 Covariates

Self-reported socio-demographic characteristics regarding age, gender, race, education level, marital status and family income were collected for each participant. Other data was collected including smoking status, alcohol use, diabetes, hypertension (identified as a self-report of a doctor’s diagnosis, systolic blood pressure ≥ 140 mmHg, or diastolic blood pressure ≥ 90 mmHg) (19), hypercholesterolemia. Smoking status were subdivided as never (smoked less than 100 cigarettes in life and not currently smoking), former (smoked at least 100 cigarettes in life and not currently smoking), and active smoker (smoked at least 100 cigarettes in life and currently smoking) (20). Alcohol use was subdivided as never, moderate, heavy, or binge according to definitions from the National Institute on Alcohol Abuse and Alcoholism in the National Institute of Health. Blood examinations were also included as some of the parameters indicated the level of systemic inflammation: white blood cell count (1000 cells/uL), segmented neutrophils number (1000 cell/uL), lymphocyte number (1000 cells/uL), hematocrit(%), platelet count(1000 cell/uL), and total cholesterol (mg/dL) and hemoglobin A1c (Hba1c)(%).

All of the variables listed above were potential confounders that might influence periodontitis. Not all of the participants provided valid data for each of these covariates and there were fewer than 5% missing values. However, direct exclusion may lead to a decrease in sample size and unpredictable bias. Therefore, we used the R package missForest, which is based on the random forest algorithm, for imputation of categorical and continuous variables (21).

### 2.5 Statistical analysis

Statistical analyses were performed with STATA MP and Rstudio. The level of significance was set at 5%. Individual sample weights were determined using the dietary day one sample weight (WTDRD1) records, to enable extrapolation to the entire noninstitutionalized U.S. population (22). Characteristics grouped by periodontitis status were presented as mean ± SE for continuous variables, and percentage(%) for categorical variables. The baseline characteristics were compared using the weighted linear regression for continuous variables, and the weighted chi-square test for categorical variables (23). Variables with significant association (*P*< 0.05) to periodontitis (**Supplementary Table 1**) were incorporated into the multivariable analyses (24).

Multivariable logistic regression models were built to evaluate the association between moderate or severe periodontitis (versus no or mild periodontitis), mean PPD, mean CAL, the number of teeth and the CDAI. Three progressively adjusted models were further developed based on the initial crude model. Model 1 was adjusted for age, race, gender, family income and education level; Model 2 was adjusted for covariates in Model 1 plus variables of systemic condition including HbA1c level and hypercholesterolemia. Model 3 was the fully adjusted model, which was adjusted for covariates in Model 2 along with smoking status. To investigate the impact of CDAI on periodontal health in different populations, we conducted a subgroup analysis of the primary outcome. We used the crude and adjusted model (Model 2) to compare between people who were non-smokers, former smokers, or active smokers.

For further investigating the association between the six micronutrients and periodontitis, we used restricted cubic splines (RCS) with 3 knots to flexibly model the association between CDAI and periodontitis, with the abnormal values in each type of micronutrients excluded. In this part, the participants were divided into two groups: no or mild periodontitis, and moderate or severe periodontitis. The association between micronutrient and periodontitis was adjusted according to Model 3. Logistic regression models were also established to explore their linear relationship (**Supplementary Table 2**).

## 3 Results

### 3.1 Characteristics of the study population

**Figure 1:**
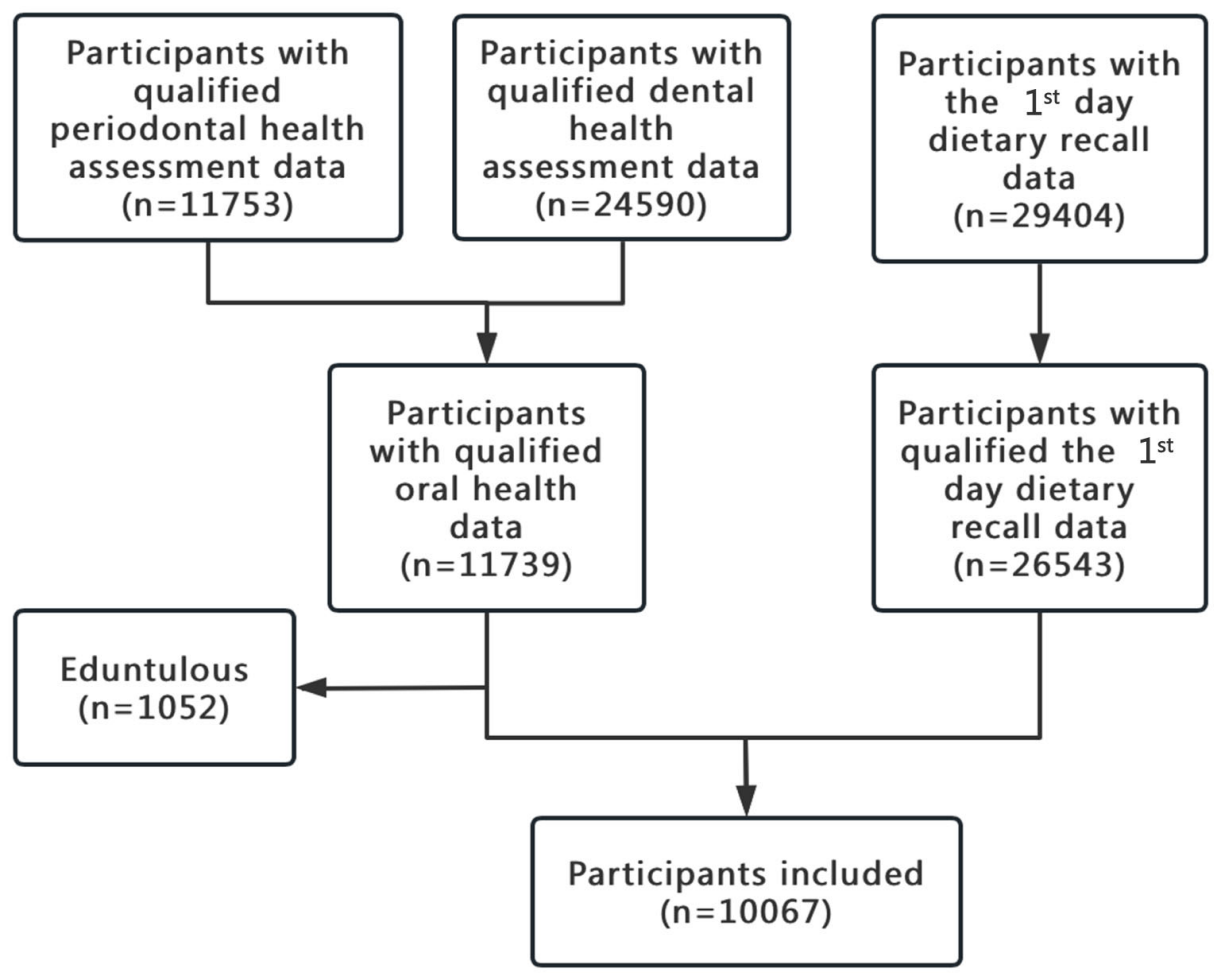
The work flow of sample selection from NHANES 2009–2014.

Among the 30,468 participants, 20,401 were excluded due to unqualified data either on oral health or the 1^st^ day dietary recall. The flowchart of participants inclusion was shown in **Figure 1**. Finally, 10,067 participants were included in the present study.

The 10,067 participants were divided into three groups based on the severity of periodontitis. The weighted prevalence was 63.4% for healthy or mild periodontitis, 28.8% for moderate periodontitis, and 7.8% for severe periodontitis. 71.5% of the participants were male in the severe-periodontitis group, while over 50% of the participants were female for the healthy or mild periodontitis group. Compared to the participants with healthy or mild periodontitis, those with moderate or severe periodontitis were more characterized by the features including being older, being Mexican American or non-Hispanic Black, having lower education levels (below undergraduate), living alone (never having been married, divorced or separated), having a lower household income (under $55,000 per year), being smokers or drinkers, or having lower CDAI (**Table 1**).

**Table 1:** Characteristics of the participants.

Characteristics	Overall	No/Mild Periodontitis	Moderate Periodontitis	Severe Periodontitis	*P* value
**Number**	10,067	5,597	3,367	1,103	
**Weighted Number**	441,400,000(100.0%)	280,000,000(63.4%)	127,000,000(28.8%)	34,400,000(7.8%)	
Continuous variables, mean ± SE					
**White Blood Cell (1000 cell/uL)**	7.126 ± 0.029	6.977 ± 0.036	7.294 ± 0.054	7.716 ± 0.119	<0.001**
**Age (year)**	50.9740.169	48.565 ± 0.215	55.317 ± 0.301	54.545 ± 0.476	<0.001**
**Lymphocyte Number (1000 cells/uL)**	2.0880.01	2.069 ± 0.012	2.115 ± 0.019	2.145 ± 0.034	0.003*
**Segmented Neutrophils (1000 cell/uL)**	4.2470.023	4.139 ± 0.028	4.364 ± 0.043	4.695 ± 0.099	<0.001**
**Hematocrit (%)**	41.390.055	41.214 ± 0.071	41.532 ± 0.101	42.301 ± 0.179	<0.001**
**Platelet (1000 cell/uL)**	237.2520.823	238.258 ± 1.021	235.204 ± 1.512	236.63 ± 3.382	0.068
**HbA1c (%)**	5.6830.011	5.562 ± 0.012	5.868 ± 0.023	5.995 ± 0.053	<0.001**
**Total Cholesterol (mg/dL)**	5.150.015	5.161 ± 0.02	5.138 ± 0.027	5.101 ± 0.05	0.256
**Tooth Number**	24.6030.077	25.755 ± 0.082	22.899 ± 0.162	21.525 ± 0.321	<0.001**
**Clinical Attachment Loss (mm)**	1.6920.013	1.248 ± 0.01	2.12 ± 0.02	3.722 ± 0.064	<0.001**
**Probing Pocket Depth (mm)**	1.6440.008	1.389 ± 0.007	1.88 ± 0.013	2.842 ± 0.035	<0.001**
Categorical variables, percentage					
**Gender (female)**	0.512	0.567	0.451	0.285	<0.001**
**Race**					<0.001**
Mexican American	0.080	0.061	0.110	0.127	
Other Hispanic	0.054	0.051	0.058	0.060	
Non-Hispanic White	0.689	0.740	0.621	0.522	
Non-Hispanic Black	0.107	0.083	0.131	0.208	
Other Race	0.071	0.065	0.080	0.083	
**Education Level (college or above)**	0.641	0.720	0.536	0.391	<0.001**
**Marital Status (live with someone)**	0.685	0.717	0.637	0.601	<0.001**
**High Income (more than $54,999 per year)**	0.559	0.648	0.418	0.353	<0.001**
**Alcohol Use**					<0.001**
Never	0.127	0.117	0.155	0.111	
Moderate	0.402	0.436	0.360	0.287	
Heavy	0.236	0.264	0.192	0.180	
Binge	0.234	0.184	0.293	0.422	
**Smoking Status**					<0.001**
Never	0.557	0.628	0.458	0.345	
Former Smoker	0.267	0.250	0.302	0.276	
Active Smoker	0.176	0.123	0.239	0.378	
**Diabetes**					<0.001**
No	0.830	0.859	0.772	0.809	
Prediabetes	0.076	0.073	0.087	0.056	
Yes	0.094	0.068	0.141	0.134	
**Have Hypertension**	0.408	0.359	0.488	0.502	<0.001**
**Have Hypercholesterolemia**	0.403	0.386	0.456	0.350	<0.001**
**CDAI**					<0.001**
1^st^	0.250	0.233	0.279	0.284	
2^nd^	0.250	0.248	0.249	0.267	
3^rd^	0.250	0.259	0.238	0.223	
4^th^	0.250	0.260	0.233	0.226	

*Indicates *P* value< 0.05; **indicates *P* value< 0.001. SE, Standard Error; HbA1c, glycated hemoglobin A1c; CDAI, composite dietary antioxidant index.

### 3.2 Association of the CDAI and periodontal outcomes

**Table 2** showed the association between CDAI and periodontal outcomes. In the unadjusted multivariable logistic regression model, a higher CDAI was associated with decreased prevalence of moderate and severe periodontitis, increased number of remaining teeth, reduced PPD and CAL. In comparison to the CDAI-1^st^ group, the CDAI-4^th^ group had significantly lower risk of moderate (odd ratio [OR] = 0.747, 95% confidence interval [CI]: 0.63–0.886, *P* = 0.001) and severe (OR = 0.712, 95% CI: 0.54–0.939, *P* = 0.016) periodontitis according to the non-adjusted model, with the risks reduced by 25.3% and 28.8%, respectively. After adjustments for the characteristics of demographics and systemic conditions in Model 2, the multivariable regression analysis still demonstrated a significant association between CDAI and severe periodontitis (OR = 0.663, 95% CI: 0.491–0.896, *P* = 0.008) in the CDAI-4^th^ group. The association between PPD, CAL and the CDAI was similar to that between periodontitis and CDAI. Furthermore, we found that the CDAI showed a stronger association with CAL than with PPD across all the four models. The CDAI was also significantly and positively correlated with the number of remaining teeth.

**Table 2:** The risk of periodontitis for the four CDAI groups indicated by the four models (N =10,067).

		Non-Adjusted	Model 1	Model 2	Model 3
Periodontitis					
Moderate	CDAI-1^st^	Reference	Reference	Reference	Reference
	CDAI-2^nd^	0.839 (0.712, 0.991)0.039*	0.872 (0.728, 1.045)0.138	0.871 (0.726, 1.044)0.136	0.918 (0.763, 1.103)0.361
	CDAI-3^rd^	0.768 (0.649, 0.908)0.002*	0.82 (0.682, 0.985)0.034*	0.82 (0.682, 0.986)0.035*	0.864 (0.717, 1.041)0.125
	CDAI-4^th^	0.747 (0.63, 0.886)0.001*	0.792 (0.654, 0.96)0.017*	0.787 (0.65, 0.955)0.015*	0.845 (0.697, 1.024)0.086
Severe	CDAI-1^st^	Reference	Reference	Reference	Reference
	CDAI-2^nd^	0.884 (0.687, 1.137)0.335	0.922 (0.7, 1.215)0.563	0.915 (0.694, 1.207)0.531	0.995 (0.747, 1.324)0.970
	CDAI-3^rd^	0.707 (0.546, 0.915)0.008*	0.738 (0.555, 0.979)0.035*	0.731 (0.55, 0.969)0.030*	0.788 (0.587, 1.059)0.114
	CDAI-4^th^	0.712 (0.54, 0.939)0.016*	0.677 (0.499, 0.918)0.012*	0.663 (0.491, 0.896)0.008*	0.728 (0.534, 0.992)0.044*
Number of Teeth					
CDAI-1^st^		Reference	Reference	Reference	Reference
CDAI-2^nd^		1.281 (0.831, 1.731)<0.001**	0.878 (0.469, 1.286)<0.001**	0.872 (0.464, 1.279)<0.001**	0.741 (0.339, 1.143)<0.001**
CDAI-3^rd^		1.796 (1.349, 2.242)<0.001**	1.077 (0.668, 1.487)<0.001**	1.059 (0.65, 1.469)<0.001**	0.912 (0.508, 1.316)<0.001**
CDAI-4^th^		2.16 (1.728, 2.592)<0.001**	1.171 (0.758, 1.584)<0.001**	1.167 (0.754, 1.58)<0.001**	0.979 (0.573, 1.385)<0.001**
PPD					
CDAI-1^st^		Reference	Reference	Reference	Reference
CDAI-2^nd^		-0.053 (-0.098, -0.008)0.022*	-0.031 (-0.073, 0.011)0.150	-0.032 (-0.073, 0.01)0.141	-0.016 (-0.058, 0.026)0.449
CDAI-3^rd^		-0.07 (-0.115, -0.025)0.002*	-0.044 (-0.086, -0.001)0.043*	-0.045 (-0.087, -0.003)0.035*	-0.027 (-0.069, 0.015)0.204
CDAI-4^th^		-0.07 (-0.114, -0.025)0.002*	-0.075 (-0.118, -0.032)0.001*	-0.076 (-0.119, -0.033)< 0.001**	-0.054 (-0.096, -0.012)0.011*
CAL					
CDAI-1^st^		Reference	Reference	Reference	Reference
CDAI-2^nd^		-0.129 (-0.206, -0.052)0.001*	-0.091 (-0.16, -0.022)0.010*	-0.091 (-0.16, -0.022)0.010*	-0.059 (-0.127,0.01)0.094
CDAI-3^rd^		-0.178 (-0.254, -0.102)<0.001**	-0.115 (-0.183, -0.047)0.001*	-0.115 (-0.183, -0.047)0.001*	-0.077 (-0.144, -0.011)0.023*
CDAI-4^th^		-0.194 (-0.265, -0.123)<0.001**	-0.154 (-0.223, -0.086)<0.001**	-0.156 (-0.224, -0.088)<0.001**	-0.109 (-0.175, -0.043)0.001*

*Indicates *P* value< 0.05; **indicates *P* value< 0.001. CDAI, composite dietary antioxidant index; PPD, probing pocket depth; CAL, clinical attachment loss. Model 1 adjusted for age, race, gender, family income and education level; and Model 2 adjusted for age, race, gender, family income, education level, HbA1c level and hypercholesterolemia; and Model 3 adjusted for age, race, gender, family income, education level, HbA1c level, hypercholesterolemia and smoking status.

### 3.3 Smoking status had impact on the association between CDAI and periodontal outcomes

When smoking was included as a covariate in the model, as mentioned in Model 3, the protective effect of CDAI on periodontitis became ambiguous. However, the coefficient of logistic regression changed by approximately 10% when compared with that in Model 2. And multifactorial analysis revealed that smoking status had a significant effect on periodontitis (**Supplementary Table 1**). Therefore, we performed subgroup analyses to explore the potential influence of smoking status on the association between CDAI and periodontitis.

As shown in **Table 3**, CDAI lost the protective effect on periodontitis in active smokers, and even in the former smokers (*P* > 0.05). Among the non-smokers, the risk of severe periodontitis for the CDAI-4^th^ group reduced by 56.6% (OR = 0.434, 95% CI: 0.291–0.649, *P<* 0.001), compared with the CDAI-1^st^ group. Similar results were found for moderate periodontitis, with the risk reduced by 34.2% (OR = 0.658, 95% CI: 0.523–0.829, *P*< 0.001). The impact of smoking on the periodontal protective role of CDAI was further investigated by the model adjusted for all of the characteristics except smoking status (Model 2). In this model, the CDAI showed protective effect only among non-smokers for severe periodontitis (OR = 0.458, 95% CI: 0.289–0.728, *P* = 0.001).

**Table 3:** Multi-regression analysis of the association between CDAI and periodontal outcomes stratified according to smoking status.

		Never Smoker		Former Smoker		Active Smoker	
		Non-Adjusted	Adjusted	Non-Adjusted	Adjusted	Non-Adjusted	Adjusted
Periodontitis							
Moderate	CDAI-1^st^	Reference	Reference	Reference	Reference	Reference	Reference
	CDAI-2^nd^	0.891 (0.717,1.108)0.302	1.014 (0.797,1.289)0.910	1.007 (0.717,1.415)0.967	0.992 (0.687,1.432)0.964	0.713 (0.481,1.058)0.092	0.694 (0.449,1.071)0.099
	CDAI-3^rd^	0.66 (0.523,0.832)<0.001**	0.796 (0.615,1.03)0.084	1.103 (0.795,1.531)0.558	1.127 (0.791,1.606)0.508	0.868 (0.581,1.298)0.491	0.78 (0.504,1.208)0.266
	CDAI-4^th^	0.658 (0.523,0.829)<0.001**	0.79 (0.607,1.027)0.078	0.902 (0.644,1.262)0.548	0.962 (0.664,1.392)0.835	1.184 (0.794,1.766)0.408	1.005 (0.646,1.565)0.982
Severe	CDAI-1^st^	Reference	Reference	Reference	Reference	Reference	Reference
	CDAI-2^nd^	0.753 (0.511,1.108)0.151	0.85 (0.553,1.305)0.456	0.814 (0.501,1.323)0.407	0.81 (0.473,1.385)0.442	1.401 (0.867,2.266)0.169	1.323 (0.766,2.286)0.316
	CDAI-3^rd^	0.609 (0.408,0.908)0.015*	0.706 (0.451,1.105)0.128	0.748 (0.446,1.252)0.269	0.777 (0.446,1.355)0.374	1.138 (0.694,1.863)0.609	0.9 (0.506,1.603)0.722
	CDAI-4^th^	0.434 (0.291,0.649)<0.001**	0.458 (0.289,0.728)0.001*	0.766 (0.421,1.395)0.384	0.742 (0.396,1.394)0.354	1.772 (1.1,2.855)0.019*	1.252 (0.738,2.125)0.404
Number of Teeth							
CDAI-1^st^		Reference	Reference	Reference	Reference	Reference	Reference
CDAI-2^nd^		1.049 (0.569,1.53)<0.001**	0.574 (0.143,1.004)0.009*	1.209 (0.065,2.352)0.038*	0.944 (-0.087,1.976)0.073	1.516 (0.459,2.573)0.005*	1.061 (0.083,2.039)0.034*
CDAI-3^rd^		1.715 (1.243,2.186)<0.001**	0.818 (0.382,1.254)<0.001**	2.087 (1.075,3.099)<0.001**	1.563 (0.63,2.495)0.001*	0.822 (-0.512,2.156)0.227	0.34 (-0.813,1.493)0.563
CDAI-4^th^		2.024 (1.595,2.454)<0.001**	0.852 (0.444,1.259)<0.001**	2.683 (1.618,3.748)<0.001**	1.639 (0.626,2.652)0.002*	0.896 (-0.312,2.105)0.146	0.465 (-0.663,1.592)0.419
PPD							
CDAI-1^st^		Reference	Reference	Reference	Reference	Reference	Reference
CDAI-2^nd^		-0.036 (-0.091,0.019)0.199	-0.012 (-0.063,0.039)0.653	-0.033 (-0.125,0.059)0.482	-0.032 (-0.119,0.054)0.461	-0.014 (-0.136,0.109)0.826	-0.022 (-0.136,0.092)0.702
CDAI-3^rd^		-0.074 (-0.127, -0.021)0.007*	-0.038 (-0.088,0.013)0.142	-0.038 (-0.128,0.052)0.404	-0.04 (-0.126,0.046)0.364	0.048 (-0.078,0.175)0.451	0.009 (-0.114,0.131)0.887
CDAI-4^th^		-0.1 (-0.151, -0.049)<0.001**	-0.089 (-0.139, -0.039)<0.001**	-0.023 (-0.116,0.07)0.623	-0.042 (-0.133,0.048)0.360	0.122 (0.002,0.242)0.047*	0.022 (-0.091,0.135)0.706
CAL							
CDAI-1^st^		Reference	Reference	Reference	Reference	Reference	Reference
CDAI-2^nd^		-0.094 (-0.17, -0.019)0.015*	-0.042 (-0.109,0.025)0.222	-0.114 (-0.3,0.072)0.230	-0.105 (-0.275,0.065)0.226	-0.058 (-0.278,0.162)0.606	-0.066 (-0.263,0.131)0.513
CDAI-3^rd^		-0.188 (-0.256, -0.12)<0.001**	-0.094 (-0.154, -0.034)0.002*	-0.15 (-0.322,0.023)0.088	-0.123 (-0.282,0.036)0.129	0.061 (-0.184,0.305)0.626	-0.015 (-0.236,0.206)0.896
CDAI-4^th^		-0.203 (-0.269, -0.137)<0.001**	-0.126 (-0.189, -0.064)<0.001**	-0.189 (-0.354, -0.024)0.025*	-0.134 (-0.293,0.025)0.099	0.095 (-0.12,0.311)0.387	-0.074 (-0.272,0.124)0.465

*Indicates *P* value< 0.05; **indicates *P* value< 0.001. CDAI, composite dietary antioxidant index; PPD, probing pocket depth; CAL, clinical attachment loss. Adjusted model, namely Model 3, adjusted for age, race, gender, family income, education level, HbA1c level, hypercholesterolemia and smoking status.

Similar results were found regarding the association of CDAI with mean PPD and mean CAL in Model 2. For the non-smokers in the CDAI-4^th^ group, the CDAI were significantly associated with PPD values (weighted β[SE] = -0.089[0.026], *P<* 0.001) and CAL values (weighted β[SE] = -0.126[0.031], *P<* 0.001). For the active smokers or former smokers, there were no correlations (*P* > 0.05) between PPD or CAL and CDAI. Additionally, higher CDAI were significantly associated (weighted β[SE]=1.639[0.517], *P =* 0.002) with increased number of remaining teeth among the former smokers.

### 3.4 Non-linear association between the CDAI ingredients and periodontitis

**Figure 2:**
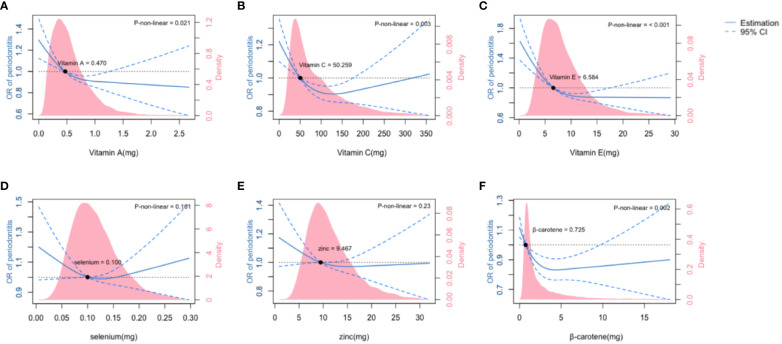
The association of six ingredients of Composite Diatery Antioxidant Index (CDAI) with moderate and severe periodontitis. The association of Vitamin A **(A)**, Vitamin C **(B)**, Vitamin E **(C)**, Selenium **(D)**, Zinc **(E)** and β-carotene **(F)** with the OR of periodontitis was shown in the restricted cubic splines, as indicated by the model adjusted for age,race,gender,family income, education level, HbA1c level, hypercholesterolemia and smoking status. The dashed line represents the 95%CI. Density plots represent the distribution of populations with consecutively varied levels of micronutrient intakes. CI, confidence interval; OR, odds ratio.

Each ingredient of the CDAI and its linear correlation with periodontitis were investigated using multivariate linear regression. Vitamin A, Vitamin E and β-carotene were found to be significantly associated with periodontitis (*P<* 0.05) in the fully adjusted model (**Supplementary Table 2**). The association between the six micronutrients and periodontitis was shown in the restricted cubic splines curves (**Figure 2**). The association of Vitamin A and Vitamin E with periodontitis assumed a L-shaped relationship (**Figures 2A, C**, *P* for non-linearity< 0.05), while Vitamin C and β-carotene had a U-shaped relationship with periodontitis (**Figures 2B, F**, *P* for non-linearity< 0.05).

We found that there were threshold levels for β-carotene, Vitamin A, C and E intakes where the risk of periodontitis significantly increased below these levels and was inversely correlated with the amount of micronutrient intakes. For Vitamin A and E, dietary intakes above the threshold levels were associated with steadily protective effect on periodontal health, within the dose range observed in this study. However, having Vitamin C and β-carotene far beyond the threshold levels tended to lead to increased risk of periodontitis. No significant association (*P* > 0.05) was observed between selenium or zinc with periodontitis.

## 4 Discussion

In light of this study, independent of factors including age, gender, race, education level, family income, living habit and systemic conditions, the stratified CDAI was significantly associated with periodontitis and tooth loss. However, the periodontal protective role of CDAI vanished for active and former smokers.

The CDAI ingredients contain essential trace elements for various proteins and enzymes that are involved in antioxidant and anti-inflammatory processes, collagen synthesis, tyrosine metabolism, and protection against cancers (16, 25). Despite the widely recognized effectiveness and accessibility of the micronutrient protecting against inflammatory diseases (15), previous studies on the associations of nutrients with the risk of periodontitis have yielded controversial findings (26–31). The discrepancies might have arisen from 1) the interactions between different nutrients being overlooked when each type of nutrient was considered separately; 2) some mild effects of the nutrients being overwhelmed by stronger influence from specific covariates (30); 3) non-linear association being underestimated in a large population (32); 4) difficulty in recognizing slight impact of micronutrient levels in the serum on periodontal disease (29). In this study, we evaluated the anti-oxidant capacity of micronutrient through dietary intake using CDAI, and investigated the impact of CDAI on periodontitis, attempting to draw a more reliable conclusion by avoiding the above defects.

Interestingly, the present study revealed stronger association of the CDAI with CAL than with PPD. Pathologically, with CAL and PPD both being the essential elements for the diagnosis of periodontitis, CAL is a more direct indicator of destruction of tooth supporting tissue including alveolar bone (33). The strong link between CDAI and CAL stressed the potential protective effect of CDAI on periodontal tissue. In the fully adjusted model (Model 3), despite vanished effect of CDAI on periodontitis-related factors including CAL and PPD, the stratified CDAI still significantly correlated with the number of remaining teeth. Since tooth loss may result from multiple causes such as caries and periodontal diseases (34), the beneficial effect of micronutrient on teeth may be derived from protection against tooth decay, as well as factors other than periodontal health (35).

It is well documented that conventional and electronic cigarette smoking are prominent risk factors for periodontitis (36, 37). Cigarette smoke contains toxic substances that dissolve in oral epithelial linings and spread throughout the body, interfering in biological events involved in redox homeostasis, immune responses, bone metabolism and tissue repair (38, 39). Tobacco-rich conditions facilitate proliferation of *Filifactor alocis* and enhance its pathogenicity (40). The detrimental effect of smoking on periodontal health persists long even after quitting. Former smokers still had a higher risk of tooth loss caused by periodontal diseases compared to the average population, and it took up to 15 years after quitting to reach the same risk level as before smoking (41). Our study lent support to this notion by demonstrating periodontal protective effect of CDAI on non-smokers but not on active or former smokers.

Previous studies had demonstrated the non-linear relations between CDAI and oxidative stress-related diseases (23, 42). The association between specific micronutrients and periodontitis was examined by using two-piecewise linear regression models (31). In the present study, we focused on dietary micronutrient intakes and looked into the six CDAI ingredients, based on models adjusted for covariates including demographics, lifestyles and systemic conditions. It is worth noting that there were threshold levels for the CDAI ingredients, having micronutrients below which was associated with increased risk of periodontitis. These threshold levels were 0.470 mg for Vitamin A, 6.584 mg for Vitamin E, 50.259 mg for Vitamin C, and 0.725 mg for β-carotene. By superimposing the density plots on the restricted cubic splines (**Figure 2**), we revealed that there were large populations whose intakes of Vitamin A, C and E were lower than the threshold levels. According to the UK National Health Service, the recommended dietary allowances (RDA) for Vitamin A, C and E were 0.6~0.7 mg, 40 mg and 3~4 mg, respectively (15). Our study confirmed the validity of these RDAs in protecting periodontal health, and yielded further suggestions that slightly higher intakes of Vitamin C and E above the RDAs might be recommended for patients accepting adjuvant therapies, and for preventive dental care for the populations at high risk of periodontitis.

Note that Vitamin intakes were not the more the better. The risk of periodontitis tended to increase again after high intakes of Vitamin C and β-carotene far beyond the threshold levels, possibly due to overdosed effect or imbalanced nutrition (43). Although with not-yet-observed overdosed effect of Vitamin A and E on periodontitis, it is of vital importance to take into consideration the whole body when making diet instructions. Overabundance of Vitamin A was known to cause liver toxicity (15).

The role of zinc and selenium on antioxidation and periodontal regeneration have been reported in previous studies (15, 44). Several studies on animal models revealed their effectiveness in ameliorating periodontitis (45, 46). However, this study showed that zinc or selenium had no significant association with the risk of periodontitis. The discrepancy might result from the differences in the delivery route (drug administration versus dietary intakes), the form of drug or food (a single drug versus a single element separately analyzed from composite nutrients), and sample selection (diseased subjects versus general populations). A previous study showed that serum zinc levels were associated with the risk of periodontitis in non-diabetic smokers but not non-smokers (47), suggesting the periodontal protective effect of zinc in specific but not general conditions. Further prospective studies like randomized controlled trails are warranted to determine the effect of specific micronutrients from dietary intakes.

The essential role of oxidative stress in pathogenesis of periodontitis has been well recognized. Free radicals contribute to tissue destruction by damaging DNA and proteins, acting as intracellular signal mediators for osteoclast activation, and causing lipid peroxidation and bone destruction (1). Antioxidants protect against oxidative stress by eliminating excessive free radicals (48). Studies have demonstrated that the components of CDAI contributed to the decrease in oxidative stress (14, 15), suggesting that the CDAI components might help strengthen systemic antioxidant defense mechanisms. Interestingly, Hung N. Luu et al. (49) presented a different conclusion that CDAI was not significantly correlated to oxidative stress. The discrepancy of the results may be caused by differences in samples and evaluation metrics. Luu’s study focused on the Chinese population, samples with different ethnic background from the United States. Additionally, in Luu’s study oxidative stress levels were measured using Urinary F2 isoprostanes and Urinary F2 isoprostane metabolites, with no consideration of indicators like malondialdehyde, glutathione, glutathione reductase etc.

Our study is the first to investigate the collective effect of dietary antioxidant intakes on periodontal health based on a large population. The NHANES used a multi-stage probability sampling process to create samples that were well representative of the noninstitutionalized population in the United States. Thus, the conclusion of this study should be applicable to the general population in the United States. Although with significant association, the cause-and-effect relationship between CDAI and periodontitis cannot be determined by this cross-sectional study. However, the effectiveness of a micronutrient-assisted nonsurgical therapy for chronic periodontitis proved by a randomized, double-blind trial (50) suggested the causal effect of micronutrient intakes on improvement of periodontal health, which was less likely to be explained by the reverse causality.

Considering the individual variability in susceptibility to periodontitis and regional difference in accessibility to dental care across various populations (51), incorporating dietary antioxidant intake into long-term prevention and adjuvant therapy may be a cost-effective and sustainable approach. In light of this study, we recommend proper micronutrient intake from food for adults. The collective effect of multiple antioxidants in a diet helps protect against periodontitis. Moreover, considering the etiological association between periodontitis and systemic diseases such as diabetes, Alzheimer’s disease and pre-eclampsia (52–54), dietary antioxidant intakes may also be beneficial for periodontitis patients who have elevated risks of developing these non-communicable diseases.

Although with interesting findings, the results of this study should be interpreted with caution. The pathogenesis of periodontitis is complicated. The effect of nutrients on periodontal health status may be direct or indirectly caused by the nutrient-correlated factors like dietary patterns, or diet-modulated general conditions like systemic diseases. Additionally, 24 hours are not long enough to give an overall and accurate reflection of individual’s dietary habit. The dietary intake record in a 24-hour timeframe is just a representative, rather than general dietary status. It is difficult and practically impossible to include all of the confounding factors during surveys. Some possible covariates like pregnancy, medications, lifestyles, oral hygiene status and systemic diseases other than what we have already covered, were of limited availability in the database. Further studies would be of interest after acquisition of extended information.

## 5 Conclusions

Our study showed that increased CDAI was associated with reduced risk of periodontitis and tooth loss. There were threshold levels for β-carotene, Vitamin A, C and E where dietary intakes above these levels were associated with better periodontal health. However, the protective effects were largely reduced and even eliminated in subjects with smoking history or current smoking status.

## Data availability statement

The original contributions presented in the study are included in the article/**Supplementary Material**. Further inquiries can be directed to the corresponding author.

## Ethics statement

Ethical review and approval was not required for the study on human participants in accordance with the local legislation and institutional requirements. Written informed consent from the [patients/participants OR patients/participants legal guardian/next of kin] was not required to participate in this study in accordance with the national legislation and the institutional requirements.

## Author contributions

ZM: Methodology, Formal analysis, Writing – original draft, Writing – review & editing. WZ: Data curation, Writing – original draft, Writing – review & editing. XM: Software, Writing – review & editing. HX: Conceptualization, Supervision, Validation, Writing – review & editing.
