# Supplementary material for: The association of composite dietary antioxidant index with periodontitis in NHANES 2009–2014
Source: Front Immunol. 2024 Jun 24;15:1384272. doi: 10.3389/fimmu.2024.1384272 (PMC11228179; doi:10.3389/fimmu.2024.1384272)
Supplement: Supplementary file 1 [file Table_1.docx]

**Supplementary Table 1.** Multifactor analysis.

|  | **Moderate Periodontitis** | **Severe Periodontitis** |
| --- | --- | --- |
| **Age (year)** | **1.051(1.045,1.057) <0.001^**^** | **1.063(1.054,1.072) <0.001^**^** |
| **White Blood Cell (1000 cell/uL)** | 1.088(0.849,1.394)0.504 | 1.393(0.931,2.085)0.107 |
| **Lymphocyte Number (1000 cells/uL)** | 0.958(0.724,1.266)0.761 | 0.717(0.451,1.139)0.159 |
| **Segmented Neutrophils (1000 cell/uL)** | 0.969(0.746,1.259)0.813 | 0.798(0.517,1.234)0.311 |
| **Hematocrit (%)** | 0.983(0.963,1.003)0.097 | 0.985(0.955,1.017)0.362 |
| **HbA1c (%)** | **1.158(1.071,1.252) <0.001^**^** | **1.328(1.178,1.496) <0.001^**^** |
| **Gender** |  |  |
| Male | Reference | Reference |
| Female | **0.481(0.399,0.579) <0.001^**^** | **0.235(0.174,0.315) <0.001^**^** |
| **Race** |  |  |
| Mexican American | **Reference** | **Reference** |
| Other Hispanic | **0.59(0.458,0.76) <0.001^**^** | **0.54(0.368,0.794)0.002^*^** |
| Non-Hispanic White | **0.405(0.331,0.497) <0.001^**^** | **0.32(0.235,0.436) <0.001^**^** |
| Non-Hispanic Black | **0.771(0.615,0.966)0.024** | **1.12(0.812,1.545)0.489** |
| Other Race | **0.792(0.605,1.036)0.088** | **0.866(0.575,1.306)0.493** |
| **Education Level (college or above)** | **0.709(0.611,0.823) <0.001^**^** | **0.463(0.363,0.591) <0.001^**^** |
| **Marital Status (live with someone)** | 0.928(0.796,1.082)0.340 | 0.841(0.661,1.07)0.159 |
| **High Income (more than $54,999 per year)** | **0.575(0.496,0.667) <0.001^**^** | **0.549(0.425,0.709) <0.001^**^** |
| **Alcohol Use** |  |  |
| Never | Reference | Reference |
| Moderate | 0.772(0.631,0.944)0.012^*^ | 0.813(0.576,1.145)0.236 |
| Heavy | 0.873(0.696,1.097)0.245 | 1.108(0.732,1.676)0.627 |
| Binge | 1.004(0.789,1.278)0.974 | 1.27(0.86,1.876)0.230 |
| **Smoking Status** |  |  |
| Never | **Reference** | **Reference** |
| Former Smoker | **1.369(1.157,1.62) <0.001^**^** | **1.453(1.09,1.936)0.011^*^** |
| Active Smoker | **2.686(2.175,3.316) <0.001^**^** | **4.362(3.172,5.998) <0.001^**^** |
| **Diabetes** |  |  |
| No | Reference | Reference |
| Prediabetes | 1.043(0.809,1.346)0.743 | 0.669(0.451,0.994)0.046**^*^** |
| Yes | 1.017(0.791,1.306)0.897 | 0.717(0.477,1.078)0.110 |
| **Have Hypertension** | 0.967(0.828,1.129)0.668 | 1.065(0.84,1.349)0.605 |
| **Have Hypercholesterolemia** | **0.872(0.75,1.015)0.078** | **0.553(0.436,0.702) <0.001^**^** |

*Indicates P value < 0.05; **indicates P value < 0.001. Abbreviations: HbA1c, glycated hemoglobin A1c.
