# Supplementary material for: The association of composite dietary antioxidant index with periodontitis in NHANES 2009–2014
Source: Front Immunol. 2024 Jun 24;15:1384272. doi: 10.3389/fimmu.2024.1384272 (PMC11228179; doi:10.3389/fimmu.2024.1384272)
Supplement: Supplementary file 2 [file Table_2.docx]

**Supplementary Table 2.** Individual micronutrients average intake level and their linear relationship with periodontitis.

|  | **mean ±SD** | **Non-Adjusted** | **Model 3** |
| --- | --- | --- | --- |
| **Vitamin A** | 75.655±1.029 | **0.673(0.577,0.783) <0.001^**^** | **0.778(0.660,0.918)0.003^*^** |
| **Vitamin C** | 10.905±0.082 | 0.999(0.998,1.000)0.012^*^ | 0.999(0.998,1.000)0.116 |
| **Vitamin E** | 0.110±0.001 | **0.968(0.955,0.981) <0.001^**^** | **0.982(0.967,0.997)0.017^*^** |
| **selenium** | 8.158±0.074 | 0.826(0.270,2.520)0.736 | 0.804(0.214,3.0227)0.746 |
| **zinc** | 0.594±0.007 | 0.993(0.982,1.004)0.185 | 0.996(0.984,1.009)0.576 |
| **β-carotene** | 2.046±0.046 | **0.948(0.926,0.971) <0.001^**^** | **0.969(0.945,0.993)0.012^*^** |

*Indicates P value < 0.05; **indicates P value < 0.001. Abbreviations: SD, Standard Deviation. Model 3 adjusted for age, race, gender, family income, education level, HbA1c level, hypercholesterolemia and smoking status.
